# Rationale and clinical application of antimicrobial stewardship principles in the intensive care unit: a multidisciplinary statement

**DOI:** 10.1186/s44158-023-00095-6

**Published:** 2023-05-01

**Authors:** Andrea Cortegiani, Massimo Antonelli, Marco Falcone, Antonino Giarratano, Massimo Girardis, Marc Leone, Federico Pea, Stefania Stefani, Bruno Viaggi, Pierluigi Viale

**Affiliations:** 1 Department of Surgical, Oncological and Oral Science, University of Palermo, Via Liborio Giuffrè 5, 90127 Palermo, Italy; 2 Department of Anaesthesia, Intensive Care and Emergency, University Hospital Policlinico Paolo Giaccone, 90127 Palermo, Italy; 3 Department of Anesthesiology and Intensive Care Medicine, Università Cattolica del Sacro Cuore, Rome, Italy; 4 Anesthesia and Intensive Care, Fondazione Policlinico Universitario A. Gemelli IRCCS, Rome, Italy; 5 Infectious Diseases Unit, Department of Clinical and Experimental Medicine, Azienda Ospedaliera Universitaria Pisana, University of Pisa, Pisa, Italy; 6 Intensive Care Unit, University Hospital of Modena, Modena, Italy; 7 Department of Anaesthesia and Intensive Care Unit, Aix-Marseille University, AP-HM, North Hospital, Marseille, France; 8 Department of Medical and Surgical Sciences, Alma Mater Studiorum-University of Bologna, 40138 Bologna, Italy; 9 Clinical Pharmacology Unit, IRCCS Azienda Ospedaliero-Universitaria Di Bologna, 40138 Bologna, Italy; 10 Microbiology Section, Dept of Biomedical and Biotechnological Science, University of Catania, Catania, Italy; 11 Unità Operativa Complessa (UOC) Laboratory Analysis, University Hospital Policlinico-San Marco, Catania, Italy; 12 Department of Anesthesiology, Neuro-Intensive Care Unit, Careggi University Hospital, 50139 Florence, Italy; 13 Department of Medical and Surgical Sciences, University of Bologna, Bologna, Italy; 14 Infectious Disease Unit, IRCCS Azienda Ospedaliero-Universitaria Di Bologna, Bologna, Italy

**Keywords:** Sepsis, Antimicrobial stewardship, Infection

## Abstract

**Background:**

Antimicrobial resistance represents a major critical issue for the management of the critically ill patients hospitalized in the intensive care unit (ICU), since infections by multidrug-resistant bacteria are characterized by high morbidity and mortality, high rates of treatment failure, and increased healthcare costs worldwide. It is also well known that antimicrobial resistance can emerge as a result of inadequate antimicrobial therapy, in terms of drug selection and/or treatment duration. The application of antimicrobial stewardship principles in ICUs improves the quality of antimicrobial therapy management. However, it needs specific considerations related to the critical setting.

**Methods:**

The aim of this consensus document gathering a multidisciplinary panel of experts was to discuss principles of antimicrobial stewardship in ICU and to produce statements that facilitate their clinical application and optimize their effectiveness. The methodology used was a modified nominal group discussion.

**Conclusion:**

The final set of statements underlined the importance of the specific interpretation of antimicrobial stewardship’s principles in critically ill patient management, quasi-targeted therapy, the use of rapid diagnostic methods, the personalization of antimicrobial therapies’ duration, obtaining microbiological surveillance data, the use of PK/PD targets, and the use of specific indicators in antimicrobial stewardship programs.

## Background

Antimicrobials are used in intensive care units (ICUs) on a global scale [1]. It has been estimated that more than 70% of critically ill patients receive antibiotics during their hospital stay [2] and that antimicrobial consumption in ICUs is as much as 10 times higher than in conventional wards [3]. This is in line with the high incidence of infections in this setting, where susceptibility to infectious risk is 5–10 times higher than in the hospital and community case mix [4].

Excessive use of antimicrobials in ICUs contributes to the emergence of multiresistance [5, 6]. This phenomenon is also related to the routine adoption of empirical therapies, based on the use of broad-spectrum antibiotics [7]. This is common in critical care settings, where rapid interventions are required, often carried out in the absence of definitive microbiological information, in terms of isolated species and phenotypic or genotypic chemosensitivity [2].

Infections sustained by multidrug-resistant bacteria (MDRO) increase the severity of infectious complications, negatively affecting morbidity, mortality, and care costs [8, 9].

The higher incidence of infections and antimicrobial resistance in ICUs depends on the unavoidable presence of numerous variables associated with infectious risk, among which are the use of invasive devices, the advanced age of patients, the presence of immunosuppression, the prolonged period of hospitalization and/or invasive mechanical ventilation, and the administration of antibiotic therapies, often protracted over time and the use of broad-spectrum drugs [10].

Literature data claims that MDRO infections, which are increasingly common in both community and hospital settings [11–15], are the most common cause of inappropriate antibiotic therapy. Indeed, infections by MDRO are association also to a longer delay to an appropriate antibiotic treatment, when achieved [8], defined on antibiotic susceptibility of the pathogen and adequate tissue penetration for the source of infection. In this regard, several studies claim that up to 60% of prescriptions in ICUs are inappropriate [12, 16, 17] and that this is associated with unfavorable outcomes [18–21]. In particular, there is evidence of increased mortality among patients infected by MDRO [22–27].

However, the concept of inappropriate therapy should extend beyond the scope of chemosensitivity and should include the patient’s pathophysiological condition and the pharmacokinetic/dynamic characteristics of the antimicrobials. In this respect, incorrect dosages and/or modes of administration inconsistent with the properties of the different compounds could also play a significant role [28].

Indeed, in critically ill patients, clinical characteristics and pathophysiological alterations (i.e., increased vascular permeability in septic shock, acute kidney injury, hepatic dysfunction) can have a marked effect on drug exposure levels, leading to significant variability in efficacy in vivo. Thus, the choice of the appropriate drug and dosage is pivotal to reduce the risk of therapeutic failure, on the one hand, and the risk of toxicity, on the other [29].

The timing, when starting appropriate antibiotic therapy, has also proved crucial: several studies have shown a correlation between treatment delay and increased mortality in critically ill patients [30–32]. One observational study emphasized that the timing of the start of appropriate antibiotic therapy is a crucial element in the proper management of patients with MDRO. For example, in patients admitted to ICUs with carbapenemase-producing *Klebsiella pneumoniae* (KPC) bacteremia, a delay in the administration of antibiotic therapy, which is active against this organism, is associated with higher mortality [33]. It is therefore necessary, especially in ICUs, to implement strategies to identify patients at higher risk of infection with multi-resistant germs at an early stage, to optimize the choice of empirical antibiotic therapy, decided on patients’ risk factors and rectal colonization status [34].

For the above reasons, in the critical context, there is a need to structure antimicrobial stewardship programs (ASPs), which aim to (1) optimize the use of antimicrobials [35, 36], favoring the use of therapies aimed at a more selective elimination of the microorganism responsible for the infection; (2) adapt dosages and modes of administration; and (3) contain treatment duration [18].

This integrated, multidisciplinary approach promotes the adoption of appropriate therapy (in terms of dose, duration and route of drug administration) to minimize the risk of developing antimicrobial resistance [37]. It makes it possible to eradicate the infection while minimizing collateral damage and the emergence of resistance [38]. Overall, its implementation in the ICU setting improves the management of high antibiotic consumption and achievable outcomes, consequently reducing the costs of therapy [39–41]. However, to make ASPs truly effective, these programs need to be implemented not only in ICUs but also in hospital wards, from which more than 60% of patients admitted to ICUs come.

Overall, appropriate antimicrobial management includes:

i) rapid identification of the pathogen and its susceptibility to antibiotics, avoiding unnecessary use of broad- spectrum antibiotics;
ii) treatment of the infection, choosing an optimized therapy based on the pharmacokinetic/pharmacodynamic characteristics, the behavior of the drug at the specific site of infection, and the patient’s pathophysiological characteristics [38];
iii) pursuit of rapid therapeutic action and the use of objective clinical and bio-humoral response parameters, aimed at avoiding prolonged therapy times.

In the context of ASPs, all the following elements must be taken into account [42]:

- Commitment by hospital leadership to provide human, financial resources.
- Identification of professionals responsible for program management and results.
- Appointment of a reference pharmacist to implement the use of antibiotics.
- Implementation of interventions (such as prospective audit, feedback, or pre-authorization)
- Monitoring the prescription of antibiotics and the impact of interventions.
- Effective communication with relevant figures, in relation to information on antibiotic use and the phenomenon of resistance.
- Educational pathways for prescribers, pharmacists, nurses, and patients, providing information on antibiotic adverse reactions, resistance, and optimal prescribing.

**Fig. 1:**
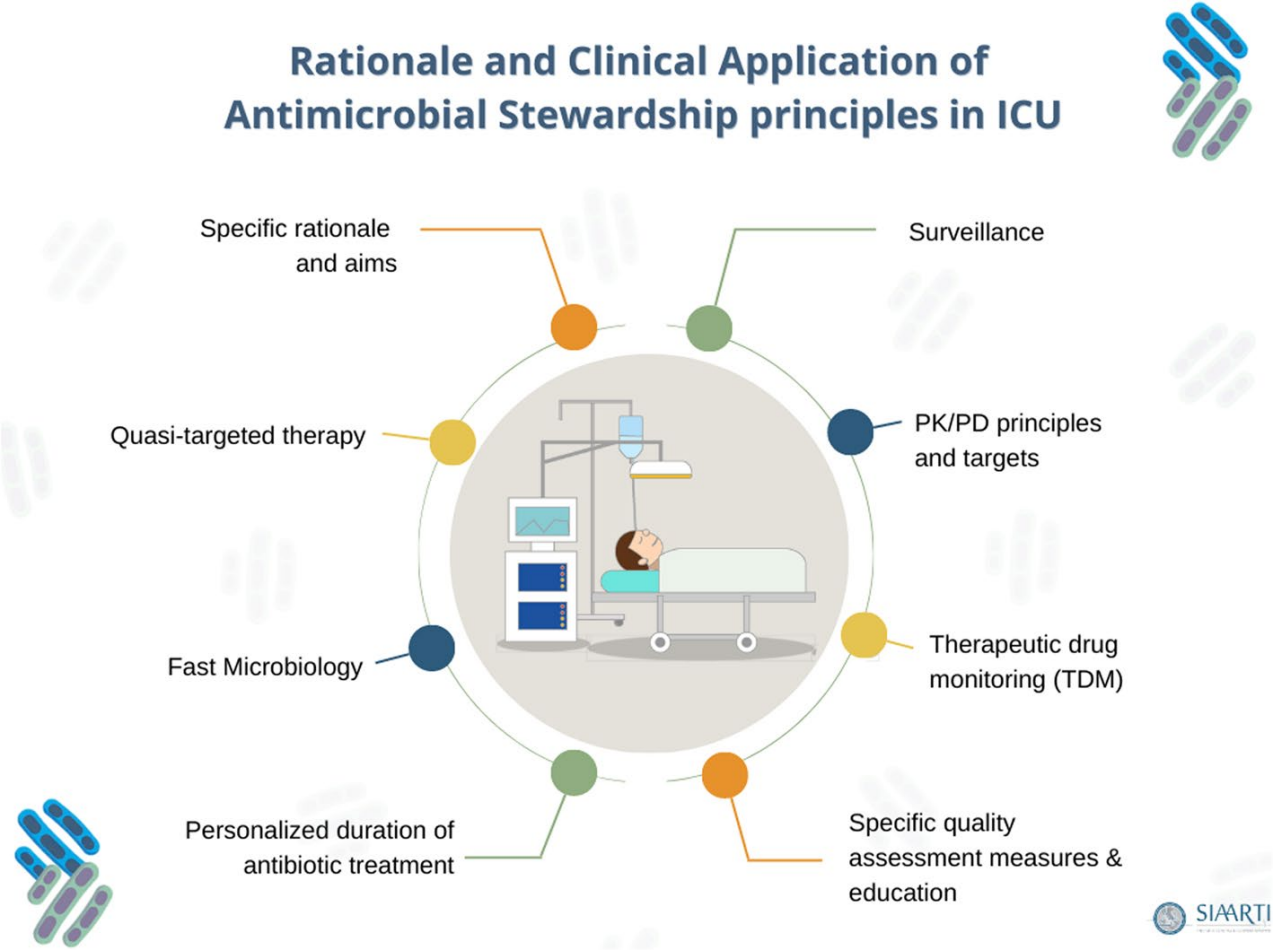
The figure shows the main components and themes related to the clinical application of antimicrobial stewardship principles in the intensive care unit

The aim of this consensus document is to discuss principles of ASP in critically ill patients in ICU and to produce statements that can facilitate their clinical application and optimize their effectiveness in this context (Fig. 1).

## Methods

The project was promoted by the Italian Society of Anesthesia Analgesia Resuscitation and Intensive Care (SIAARTI), with the participation of a multidisciplinary group of experts, anesthesiologists, infectious diseases specialists, clinical pharmacologists and clinical microbiologists, with proven expertise on issues related to critical patient infections, the phenomenon of antibiotic resistance, and ASP.

The design of the project was developed and guided by a clinician with experience in methodology (AC). Specifically, the methodology applied (modified nominal group technique (mNGT)) made it possible to generate and guide a discussion among a limited number of people on a topic and to obtain consensus on certain items (i.e., priority topics of discussion on the topic), produced by the panel participants, in a relatively short time [43]. The panel methodologist (AC) also acted as the facilitator of the mNGT.

The project’s steps included:

- Scoping workshop phase (i.e., round-robin; recording of ideas), during which the panel discussed and listed the items of the document, on a priority basis in terms of clinical relevance.

The items selected were as follows:

1. Significance of antimicrobial stewardship in the intensive care setting
2. Quasi-targeted treatment and rapid diagnostics
3. Reducing the duration of antimicrobial therapy
4. How to use prevalence data in regional/national/European surveillance
5. Antibiogram reading and report
6. PK/PD targets of antibiotics, therapeutic goals, and the role of expert interpretation of therapeutic drug monitoring (TDM) to optimize antibiotic therapy in the critical patient.
7. Training and monitoring
8. Group discussion (Round-robin), during which the experts expressed their opinions about the items, in the form of statements, and discussed collegially with all the other experts to (i) clarify the meaning; (ii) discuss the rationale; and (iii) comment in a multidisciplinary manner and possibly modify the statements, so as to reach a shared form. This phase took place in presence at the iCARE 2022 congress (Milan), in a single session, on 28 October 2022;
9. Drafting and sharing the draft document;
10. External review by an independent expert before manuscript submission;
11. Approval of the final form.

No formal literature review and evidence evaluation was performed. Thus, the statements must be considered the shared opinions of the panel and not evidence-based recommendations.

### Statements and rationales

*Statement 1*: Antimicrobial stewardship improves the management of antimicrobial therapy for critically ill patients in ICUs.

Antimicrobial stewardship interventions should be designed and conducted in relation to the clinical setting where the intervention is intended. Indeed, the same intervention has a different value depending on the contexts. Therefore, even the evaluation criteria adopted to define the effectiveness of the intervention within an ICU are different from those considered in different patients’ settings, especially if they are at lower intensity of care.

Moreover, it is important to consider that pharmaceutical expenditure in the ICU setting is inevitably higher than in conventional wards, including that of new antimicrobial drugs, due to the higher prevalence of risk factors for infections by MDRO and the severity of the clinical presentation. For this reason, the quality of an antimicrobial stewardship intervention cannot be evaluated only looking at cost savings, especially in the context of units with particularly severe or complex patients.

Instead, the application of antimicrobial stewardship in ICU must first and foremost aim to improve the quality of anti-infective therapy management, in terms of diagnostic appropriateness, selection of risk criteria, correctness of microbiological investigation and consistent choice of therapy (based on epidemiological, microbiological and pharmacokinetic/dynamic criteria). The pursuit of this objective certainly contributes to improving outcomes for critically ill patients and generates less selective pressure on microbial populations.

From the perspective of ensuring proper use of therapy, antimicrobial stewardship intervention is associated with a reasoned use of antibiotics and should not necessarily translate into a reduction in drug dosage or be strictly bound by the indications expressed in the various guidelines [44]. Undertreatment in critically ill patients is a serious conceptual error, as, if not more than overtreatment [45].

*Statement 2*: Quasi-targeted therapy, based on rapid diagnosis using rapid microbiological diagnostic techniques, is the main pathogen-oriented antimicrobial treatment option in ICU.

Quasi-targeted therapy represents an unvaluable opportunity for the antimicrobial treatment of patients in ICUs. It involves a significant implementation of rapid etiological diagnosis by using innovative techniques such as molecular microbiology with a syndromic approach and rapid phenotypic antibiogram. These tools have to be reliable, reproducible, and are aimed at reducing the turn-around-time (TAT), namely time between the moment when the sample is taken and the identification of the etiological agent. However, the interpretation of the results should always be based on multidisciplinary discussion and correct clinical-epidemiological framing. A recent consensus document produced by several scientific societies, brings together several recommendations, including the concept of the importance of rapid diagnostics in the identification and determination of antibiotic susceptibility [46]. These methods also reduce the duration of empirical antimicrobial therapy [46, 47]. The integration of a molecular and a phenotypic approach is relevant, considering that there has been a change in the current epidemiology regarding carbapenemase-resistant Enterobacterales. In fact, these organisms can produce different types of carbapenemases, including KPC and New Delhi metal-beta-lactamases (NDM), which may lead to changes in effective antibiotic therapy, as the new antibiotics (ceftazidime/avibactam, meropenem/vaborbactam and imipenem/relebactam) are active against KPC but not NDM [48]. Therefore, prior knowledge of the status of rectal colonization by microorganisms carrying gene determinants related to potential carbapenemase production may influence and guide the choice of early antibiotic therapy, especially in sepsis or septic shock.

The term “rapid microbiological diagnosis” identifies a time-limited procedure, which can be summarized as the detection of the pathogen within an interval of approximately 4 h and, if necessary, the performance of an antibiogram within 8 h. For the intensivist, it is important to be able to share with the clinical microbiology laboratory the diagnostic algorithms that reduce the time required to perform a test, considering that a coherent logistical organization is the basis for a correct multidisciplinary interpretation, with a view to antimicrobial stewardship.

The pre-analytical phase is a crucial step, often characterized by too long time needed for the transportation of the samples to the lab or inappropriate management of the specimens. These issues decrease the quality and effectiveness of the analysis.

In addition, recent data support [49, 50] the importance of collaboration between different professionals (multidisciplinary team) in interpreting data, thus reducing patient mortality [49].

*Statement 3*: “Short-term” antimicrobial therapy may reduce the chance of infection by multi-resistant bacteria.

The gut microbiome has a high intrinsic resistance to colonization by pathogenic and/or multi-resistant germs. In contrast, under conditions of dysbiosis, which occurs during exposure to antibiotics, the microbiota is much more prone to bacterial colonization, a condition that increases the risk of infection, disease, and pathogen spillover [51]. Since the biodiversity of the microbiota is restored rapidly when pharmacological pressure is suspended, it follows that a reduction in treatment time has a clear favorable role, both on the hospital system and on the individual patient.

Data from studies, conducted in different care settings (including ICUs) and assessing different sites of infection, showed that the duration of antibiotic therapy can be significantly reduced without the risk of leading to a worsening of the achievable outcome [52–55]. This concept cannot be based on a standardized model, but its applicability depends on the clinical evolution of the disease, the trend in biohumoral markers predictive of outcome, the results of microbiological follow-up and the immunological condition of the individual. However, the principle must be stated and pursued as far as possible, especially in the types of infections where there is evidence of feasibility and safety, such as severe community acquired pneumonia and bacteremia [56–58]. A reduction in treatment duration can also be achieved by optimizing the use of biomarkers that have been approved [57, 59] and by bearing in mind that short therapy can be considered in the case of infections localized in certain sites with adequate source control achieved (e.g., complicated intra-abdominal infections) [60]. Regarding ventilator-associated pneumonia (VAP), especially by Pseudomonas aeruginosa, considerable ambiguity remains in terms of risk of recurrence of infection in case of short therapy due to evidence limitations. An Individualized duration of therapy, considering prolongation of treatment durations over the first week for patients with a delayed or unfavorable clinical response to treatment seems a reasonable approach, based on available evidence [61, 62].

*Statement 4*: Surveillance data lead to more rational choices of empirical therapy.

To overcome the lack of representativeness in regional and national territories of the EARS-NET surveillance system, the use of network services was promoted by some Italian regions (i.e., ARS Toscana) [62]. Some regions have interactive dashboards to display surveillance data (network data) on an ongoing basis (https://www.qualitasiciliassr.it).

The importance of epidemiological data for the implementation of a rationale for empirical therapy has been evaluated in a small number of studies, which have however demonstrated its usefulness [46]. Monitoring local epidemiology is essential to provide the intensivist with basic information regarding most likely causes of infections and the possible patterns of resistance. This provides an information base from which to perform empirical clinical reasoning in order to select antimicrobial therapy, that has the highest probability of efficacy when used in the critical patient.

*Statement 5*: In the critical patient, antibiotics should be administered in a manner defined according to the pharmacokinetic-pharmacodynamic (PK/PD) targets of each drug class and site of infection.

In a “critical medicine” context, the activation of ASP represents an unmet need common to many realities. This approach cannot be simplified to a set of procedures based on uncritical adherence to a protocol, but involves precision therapy, “tailor-made” for each patient.

Therefore, an objective that may be positively pursued within a hospital setting is the monitoring of the concentrations of different drugs in the blood and/or at specific sites of infection (i.e., therapeutic drug monitoring (TDM)) [17, 63–65].

Since few centers currently have at their disposal a clinical pharmacologist, a regional pharmacology reference service may be identified. This hub, endowed with the professional figure of the clinical pharmacologist, could be particularly useful, acting both as a cultural reference for dosing and for therapeutic choices, and for the remote interpretation of pharmacological exposure data, produced and producible at peripheral sites. This is particularly relevant in a future perspective, since it is likely that simple and even point-of-care technologies will be available that can generate valuable data at low cost [66–68].

This organizational hypothesis was recently analyzed in a study that reaffirmed its favorable cost/benefit ratio [44]. With this in mind, it would be very useful to conduct a functional survey, aimed at investigating the actual possibility of carrying out TDM by the various centers and to verify the feasibility of hub-spoke projects for the interpretation of laboratory data.

*Statement 6*: Effective antimicrobial stewardship in ICU involves specific indicators and appropriate training.

Within an antimicrobial stewardship project, in an ICU setting, minimum detectable sets must be provided. Usually, this activity involves the collection of indicators, such as antibiotic consumption, cases of bacterial multi-resistance, and the number of infections developed per exposure time. However, there is a need to supplement these classic indicators with specific indicator sets.

For example, in cases of more severe patients, it is necessary to assess the inappropriateness of treatment from the point of view of the time of drug exposure, the number of times under/over treatment occurred, and whether or not the therapeutic target was reached (use of the PK/PD parameter optimization mode or not).

The appropriateness of the use of these indicators can be systematically verified through the use of audits, which can be performed by each individual unit and require that an analysis be conducted on an appropriate number of patients, who have received a certain treatment.

Training, which includes the implementation of educational courses for staff, also appears to be a relevant aspect in supporting proper implementation of antimicrobial stewardship. Its usefulness has emerged from previous proposed statements and also from some studies conducted [69–73].

### Insights for future research

Application of ASPs is supported by strong epidemiologic, logistic, and economic rationales, but the evidence showing improved outcomes in critically ill patients outcomes is relatively scarce. High external validity data (i.e., large multicenter prospective study or cluster-randomized trial) on the association between the application of ASPs and relevant patient clinical outcomes are still needed. Indeed, ICUs in a hospital located in peripheral or rural areas may have economic and/or logistic difficulties in applying ASPs. Remote consultations may be a potential solution, but this hypothesis should be tested in studies with adequate design. Performance of ASPs may be improved by artificial intelligence that may help in the early identification of patients with severe infection and sepsis. The effect on patients’ outcomes of diagnostic-therapeutic algorithms incorporating rapid microbiological diagnostic techniques in ICU should be tested in different settings and clinical scenarios. Indeed, in critically ill patients the benefit of rapid microbiology with or without ASP for the management of secondary infections should be better quantified. Appropriate interventional trials are needed. The appropriate timing for antibiotic administration in patients without shock with possible infection/sepsis is still object of discussion, particularly in critically ill patients with pre-existing organ dysfunction. Recently, a trial protocol including most of the main components of ASPs in ICU was proposed underlying the need for testing ASPs components as a unique intervention [74]. Indeed, patients with septic shock in early phase would be randomized in a cluster randomized, multicenter, trial to a personalized management strategy including rapid microbiological identification with adaptation of antibiotic dosages according to the daily TDM based on real MIC of the identified isolates versus standard care. Although (relatively) futuristic, even this comprehensive strategy may fail in improving outcomes in case of lack of knowledge on the principles of appropriate infection management in critically patients by the whole ICU team and multidisciplinary input. These pivotal elements must be improved in every ICU through quality improvement programs.

## Conclusions

Antimicrobial stewardship in the ICU involves specific rationale, elements, pathways and indicators, which are partially different from those made for different hospital settings and non-critical patients. The application of antimicrobial stewardship in the ICU improves the quality of antimicrobial therapy management, as it promotes appropriate diagnosis, more agile identification of the etiological agent and personalized treatment choice.

In the statements, the importance of early therapeutic intervention is emphasized, which is achieved through the use of rapid diagnostic techniques and the sharing of diagnostic algorithms enabling faster pathogen identification. It is also important to initiate collaborations between different professionals and to have a multidisciplinary team to interpret the microbiological results.

During the discussion, the need to initiate short duration treatment, avoiding under-treatment and favoring a quasi-targeted therapy approach, optimized according to the patient and his or her condition, emerged. Indeed, in this type of patients, the pathophysiological conditions cause pharmacokinetic changes that must be taken into account though an optimization of the PK/PD parameters. The latter can be pursued by applying correct choices in terms of antimicrobial choice, dose and administration strategy (e.g., extended or continuous infusions of beta-lactams after bolus injection), as well as through TDM, which requires the presence of an analysis laboratory and dedicated staff, but also through other strategies that can be implemented in any center.

An efficient antimicrobial stewardship program, carried out in an intensive care setting, requires the implementation of educational pathways for healthcare personnel to improve antimicrobial prescribing practice and infection control.

## Data Availability

Not applicable.

## Abbreviations

ASPs: Antimicrobial stewardship programs
AST: Antimicrobial stewardship
ICU: Intensive care unit
KPC: *Klebsiella pneumoniae* carbapenemase
MDRO: Multi-drug-resistant organisms
NDM: New Delhi metallo-β-lactamase
NP: *Nosocomial pneumonia*
PK/PD: Pharmacokinetics/pharmacodynamics
TAT: Turnaround time
TDM: Therapeutic drug monitoring
VAP: Ventilator-associated pneumoniae
